# Cerebral oxygenation during immediate fetal-to-neonatal transition and fidgety movements between six to 20 weeks of corrected age: An ancillary study to the COSGOD III trial

**DOI:** 10.1007/s00431-024-05711-3

**Published:** 2024-08-10

**Authors:** Christina Helene Wolfsberger, Bernhard Schwaberger, Berndt Urlesberger, Anna Scheuchenegger, Alexander Avian, Marlene Hammerl, Ursula Kiechl-Kohlendorfer, Elke Griesmaier, Gerhard Pichler

**Affiliations:** 1 Division of Neonatology, Department of Pediatrics and Adolescent Medicine, Medical University of Graz, Graz, Austria; 2 Research Unit for Neonatal Micro- and Macrocirculation, Department of Pediatrics and Adolescent Medicine, Medical University of Graz, Graz, Austria; 3 Institute for Medical Informatics, Statistics and Documentation, Medical University of Graz, Graz, Austria; 4 Department of Pediatrics II, Medical University of Innsbruck, Innsbruck, Austria

**Keywords:** Preterm neonates, Immediate neonatal transition, Near infrared spectroscopy, Cerebral oxygen saturation, General movement assessment, Fidgety movements

## Abstract

Fidgety movements provide early information about a potential development of cerebral palsy in preterm neonates. The aim was to assess differences in the combined outcome of mortality and fidgety movements defined as normal or pathological in very preterm neonates according to the group allocation in the randomised-controlled multicentre COSGOD III trial. Preterm neonates of two centres participating in the COSGOD III trial, whose fidgety movements were assessed as normal or pathological at six to 20 weeks of corrected age, were analysed. In the COSGOD III trial cerebral oxygen saturation (crSO_2_) was measured by near-infrared spectroscopy (NIRS) during postnatal transition and guided resuscitation in preterm neonates randomised to the NIRS-group, whereby medical support was according routine, as it was also in the control group. Fidgety movements were classified in normal or abnormal/absent at six to 20 weeks of corrected age. Mortality and fidgety movements of preterm neonates allocated to the NIRS-group were compared to the control-group. Normal outcome was defined as survival with normal fidgety movements. One-hundred-seventy-one preterm neonates were included (NIRS-group *n* = 82; control-group *n* = 89) with a median gestational age of 29.4 (27.4–30.4) and 28.7 (26.7–31.0) weeks in the NIRS-group and the control-group, respectively. There were no differences in the combined outcome between the two groups: 90.2% of the neonates in the NIRS-group and 89.9% in the control-group survived with normal outcome (relative risk [95% CI]; 0.96 [0.31–2.62]).

*Conclusions*: In the present cohort of preterm neonates, monitoring of crSO_2_ and dedicated interventions in addition to routine care during transition period after birth did not show an impact on mortality and fidgety movements defined as normal or pathological at six to 20 weeks corrected age.

**Table:** 

**What is Known** *• Fidgety movements display early spontaneous motoric pattern and may provide early information about a potential development of cerebral palsy in preterm neonates.*
**What is New** *• This retrospective observational study of the randomised-controlled multicentre COSGOD III trial is the first study investigating the potential influence of cerebral oxygenation guided resuscitation during postnatal transition period on combined outcome of mortality and fidgety movements up to 20 weeks of corrected age in very preterm neonates.* *• This study adds to the growing interest of assessing cerebral oxygenation, that monitoring of cerebral oxygen saturation and dedicated interventions during postnatal transition period according to the COSGOD III trial has no significant influence on mortality and fidgety movements defined as normal or pathological in very preterm neonates.*

**What is Known**

*• Fidgety movements display early spontaneous motoric pattern and may provide early information about a potential development of cerebral palsy in preterm neonates.*

**What is New**

*• This retrospective observational study of the randomised-controlled multicentre COSGOD III trial is the first study investigating the potential influence of cerebral oxygenation guided resuscitation during postnatal transition period on combined outcome of mortality and fidgety movements up to 20 weeks of corrected age in very preterm neonates.*

*• This study adds to the growing interest of assessing cerebral oxygenation, that monitoring of cerebral oxygen saturation and dedicated interventions during postnatal transition period according to the COSGOD III trial has no significant influence on mortality and fidgety movements defined as normal or pathological in very preterm neonates.*

## Introduction

Very premature birth is associated with a higher risk of impaired neurodevelopmental outcome following a higher rate of neonatal morbidities occurring during the neonatal period [1–4]. Early identification of abnormal neurological behaviour may have a positive impact on long-term neurodevelopmental outcome due to optimising neuroplasticity of the immature brain by accompanying therapies [5]. The Prechtl General Movement Assessment (GMA) performed until 20 weeks of corrected age is of increasing interest to provide predictive information about neonates concerning their neurodevelopment, including impaired neurodevelopmental outcome, cerebral palsy, minor neurological deficits or cognitive impairment [6–10]. General Movements (GMs) are spontaneous and complex movements that can be detected from early fetal life until four to five months of post-term age [5, 7, 11]. The repertoire of spontaneous movement patterns is described in the “global GMA” according to their age: writhing movements (after birth until six to nine weeks of corrected age) and fidgety movements (six to nine weeks until 14 to 20 weeks of corrected age).

Between six and nine weeks after birth, the writhing movements undergo a transformation into fidgety movements. Fidgety movements are characterised by circular movements with a small amplitude, moderate speed, and variable acceleration involving the neck, trunk and limbs, and can be observed until 15 to 20 weeks of corrected age. Fidgety movements can be classified as either normal or pathological. Pathological fidgety movements are further divided into mainly two categories: absence of fidgety movements (no observation of fidgety movements between six to 20 weeks of corrected age), or abnormal fidgety movements, which resemble normal fidgety movements but present themselves with irregular amplitude, speed and jerkiness [8, 12].

The impact of cerebral oxygenation using near-infrared spectroscopy (NIRS) during immediate transition after birth on neurological outcome has been described in two small observational studies analysing cerebral ultrasound findings [13] and GMA [14]. Analysing general movement optimality score (GMOS) as a semi-quantitative method showed impaired GMs, expressed by a lower GMOS, in association with increased burden of cerebral hypoxia within the first 15 min after birth [14].

The reduction of burden of cerebral hypoxia during the immediate transition period, that might improve neurological outcome by cerebral oxygen saturation (crSO_2_) monitoring guided resuscitation of preterm neonates, has been demonstrated by Pichler et al. 2016 in a randomised pilot feasibility trial (Cerebral Oxygen Saturation to Guide Oxygen Delivery—COSGOD II trial) [15]. In the COSGOD III trial [16], the aim was to increase survival without cerebral injury by crSO_2_ guided resuscitation. In this randomised-controlled trial, there was a non-significant difference of 4.3% in survival without cerebral injury between the intervention group and the control group.

Beside cerebral injury, various factors including perinatal asphyxia, chorioamnionitis, moderate-to-severe bronchopulmonary dysplasia (BPD) and/or prolonged invasive respiratory support, may contribute to poor neurodevelopmental outcome [17–19].

The aim of the present study was to assess combined outcome defined by mortality until 20 weeks of corrected age and fidgety movements between six to 20 weeks of corrected age in preterm neonates included in the COSGOD III trial. We hypothesised that crSO_2_ guided transition in addition to routine care during immediate fetal-to-neonatal transition in preterm neonates reduces mortality and/or leads to a more frequent appearance of normal fidgety movements, compared to preterm neonates treated according routine alone.

## Methods

### Study design

In the present retrospective observational study neonates included in the prospective randomised-controlled COSGOD III multicentre trial, conducted between October 2017 and February 2022, were eligible. The protocol [20] and the primary outcome [16] of the COSGOD III trial have already been published elsewhere.

For this retrospective observational study combined outcome, defined as mortality until 20 weeks of corrected age and fidgety movements performed between six to 20 weeks of corrected age were analysed and compared between the intervention (NIRS) group and control group of the COSGOD III trial. Out of eleven participating centres in the COSGOD III trial two centres performed GMA routinely and were therefore eligible for this analysis: Division of Neonatology, Department of Pediatrics and Adolescent Medicine, Medical University of Graz, Austria and Department of Pediatrics II, Medical University of Innsbruck, Austria. The present study, as an ancillary study to the COSGOD III trial, has been approved by the Regional Committee on Biomedical Ethics of the Medical University of Graz (EC number: 35–438 ex 22/23) and Medical University of Innsbruck (EC number: 1264/2023) and was conducted in accordance with the Declaration of Helsinki. The prospective randomised-controlled COSGOD III multicentre trial was registered at Clinical Trials (Number: NCT06105333).

### COSGOD III trial

Preterm neonates < 32 weeks of gestational age, were included in the COSGOD III trial and obtained continuous measurement of cerebral oxygenation during the first 15 min after birth. Before birth, preterm neonates were randomised either to the NIRS group or to the control group. In the intervention group (NIRS group) resuscitation was conducted in accordance with local guidelines and/or with the latest “Resuscitation Consensus Guidelines” [21, 22]. CrSO_2_ monitoring was visible to the clinical team. Provided that SpO_2_ was within targets, medical support was changed if crSO_2_ was less than the 10th centile or above the 90th centile. In the control group, crSO_2_ values were not visible to the clinical team and resuscitation was performed according to routine. The methods of the COSGOD III trial have been described in more detail elsewhere [16, 20].

### Inclusion and exclusion criteria for the present study

Centres that participated in the COSGOD III trial with available data on mortality and routine assessments of fidgety movements between six to 20 weeks of corrected age were included.

Centres with no available data on fidgety movements were excluded from analyses a priori.

### Demographic data and neonatal morbidities

Demographic data and neonatal morbidities including cerebral injury and all-cause mortality assessed in the COSGOD III trial were analysed in the present study. Cerebral injury was defined as intraventricular haemorrhage (IVH grade I-IV), or cystic periventricular leukomalacia (cystic PVL grade II-III). Further documented morbidities of the COSGOD III trial were respiratory distress syndrome (IRDS grade I-IV), culture proven sepsis, necrotizing enterocolitis (NEC), BPD defined as oxygen dependency or need of respiratory support at 36 weeks corrected age, retinopathy of prematurity (ROP ≥ grade II) and persistent ductus arteriosus (PDA) with medical and/or surgical intervention.

### Fidgety movements

Assessment of fidgety movements was performed between six to 20 weeks of corrected age by video recording of sequences of at least three minutes. The neonates were recorded after feeding, during periods of active wakefulness and lied in a supine position. The assessment had to be restarted when the neonates started crying, fussing or they were in suckling periods. Fidgety movements were documented by clinical staff trained and certified for GMA, who were blinded for the allocation of the neonate in the COSGOD III trial. Fidgety movements were stratified as either normal or pathological, whereby pathological fidgety movements were further divided into two categories: absent (no observation of fidgety movements between six to 20 weeks of corrected age) or abnormal (resembling normal fidgety movements but with irregular amplitude, speed and jerkiness) [8, 12].

### Primary outcome

The primary outcome of the present study was the combined outcome of mortality before 20 weeks of corrected age and the appearance of fidgety movements between six to 20 weeks of corrected age. Normal outcome was defined as survival with normal fidgety movements. Poor outcome was defined as mortality or absent/abnormal fidgety movements.

### Secondary outcomes

Survival without cerebral injury (IVH, cystic PVL), mortality, IRDS, sepsis, NEC, BPD, ROP and PDA were defined as secondary outcome parameters and were compared between the NIRS group and the control group in the present cohort.

### Statistics

Baseline characteristics of neonates are given with median and interquartile range for continuous data and numbers and percentages for categorical data. Comparison of baseline characteristics were done using Mann Whitney U-test for continuous data and Chi-square test or Fisher’s exact test for categorical data. To answer the hypothesis whether combined outcome (mortality and fidgety movements) differ between neonates of the NIRS group and neonates of the control group Chi-square test was used. Relative Risk (RR) with 95% confidence interval (95% CI) were calculated. Secondary parameters were compared between the two groups using Mann Whitney U-test for continuous data and Chi-square test or Fisher’s exact test for categorical data. Statistical analysis was performed using SAS 9.4 (SAS Institute Inc., Cary, NC, USA).

## Results

**Fig. 1:**
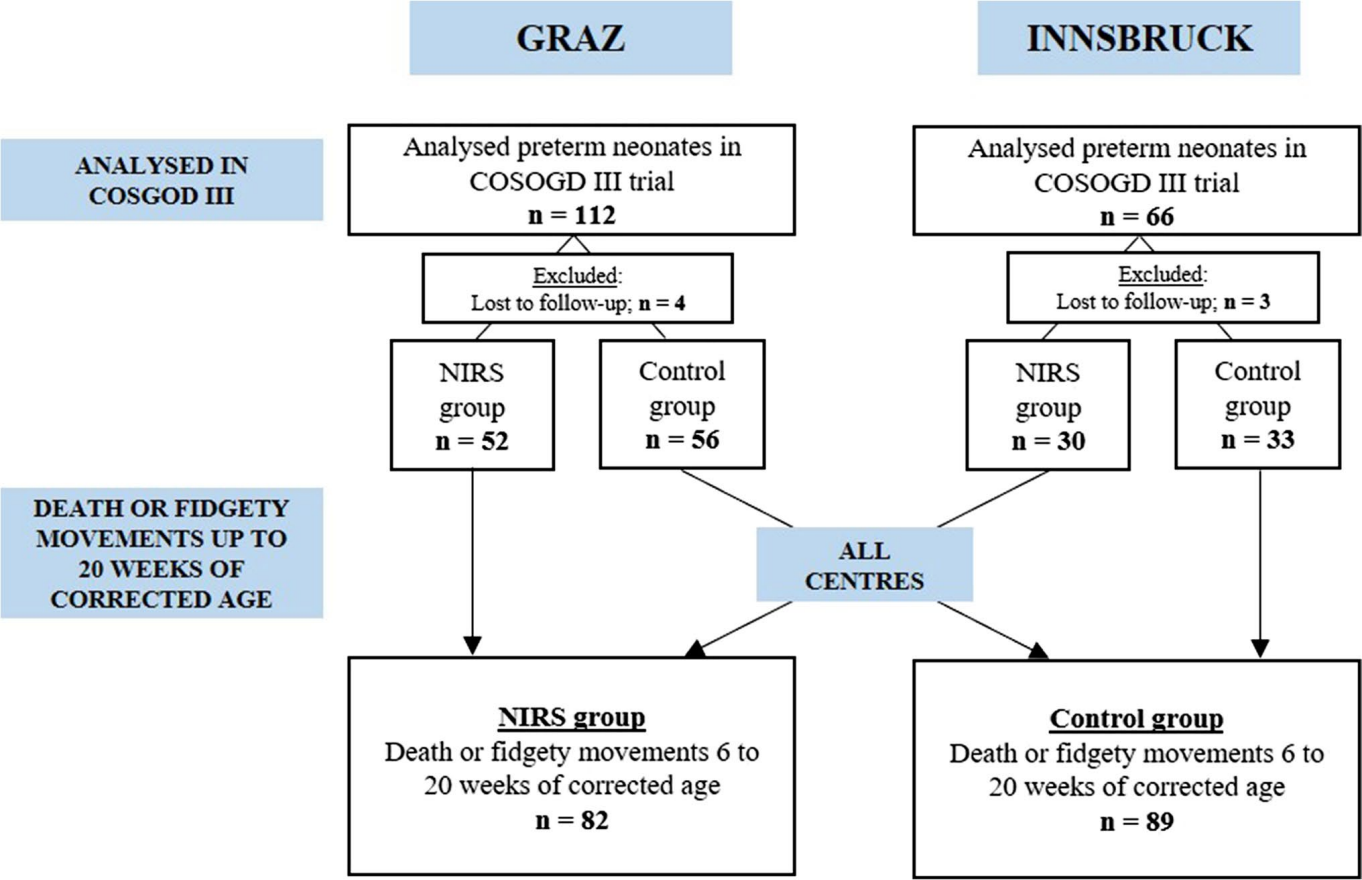
Study flow chart

In the two centres, 178 preterm neonates (Graz *n* = 112; Innsbruck *n* = 66) were included into the COSGOD III trial and were therefore eligible for the present study. Seven preterm neonates were excluded as there was no assessment of fidgety movements performed between six to 20 weeks of corrected age (Graz *n* = 4, Innsbruck *n* = 3). Thus, for the final analysis 82 preterm neonates were included in the NIRS group (Graz *n* = 52; Innsbruck *n* = 30) and 89 in the control group (Graz *n* = 56; Innsbruck *n* = 33) (Fig. 1. Study flow chart).

Demographic data of the included neonates are presented in Table 1. The median gestational age was 29.4 (27.4–30.4) weeks in the NIRS group and 28.7 (26.7–31.0) weeks in the control group. The median birth weight was 1190 (900–1450) grams and 1135 (900–1420) grams in the NIRS group and in the control group, respectively. There were no significant differences in gestational age (*p* = 0.372) and birth weight (*p* = 0.344).

**Table 1:** Neonatal characteristics and maternal and fetal causes for preterm birth of the included very preterm neonates in the NIRS group and in the control group

	NIRS group *n* = 82		Control group *n* = 89	*p*-value
Gestational age, weeks, median (IQR)	29.4 (27.4–30.4)		28.7 (26.7–31.0)	0.372
Gestational age < 28 weeks, *n* (%)	22 (26.8)		31 (34.8)	0.258
Gestational age > 28 weeks, *n* (%)	60 (73.2)		58 (65.2)	0.449
Birth weight, g, median (IQR)	1190 (900–1450)		1135 (900–1420)	0.344
Male/female, *n* (%)	46/36 (56.1/43.9)		55/34 (61.8/38.2)	0.449
Umbilical artery pH, median (IQR)	7.33 (7.28–7.37)		7.32 (7.28–7.36)	0.509
Apgar 1, median (IQR)	8.0 (6.0–8.0)		7.0 (6.0–8.0)	0.254
Apgar 5, median (IQR)	8.0 (8.0–9.0)		8.0 (8.0–9.0)	0.066
Apgar 10, median (IQR)	9.0 (9.0–9.0)		9.0 (9.0–9.0)	0.125
*Maternal causes for preterm birth*				
Antepartum bleeding, *n* (%)	12 (14.6)		4 (4.5)	0.023
Chorioamnionitis, *n* (%)	21 (25.6)		30 (33.7)	0.248
Premature rupture of membranes, *n* (%)	21 (25.6)		23 (25.8)	0.927
Preeclampsia, *n* (%)	16 (19.5)		20 (22.5)	0.635
Gestational diabetes, *n* (%)	1 (1.2)		4 (4.5)	0.370
Others, *n* (%)	16 (19.5)		26 (29.2)	0.141
*Fetal causes for preterm birth*				
Intrauterine growth restriction, *n* (%)	15 (18.3)	13 (16.9)		0.805
Fetal bradycardia, *n* (%)	14 (17.1)	11 (12.4)		0.373
Pathological doppler sonography, *n* (%)	13 (15.9)	12 (13.5)		0.373
Multiples, *n* (%)	10 (12.2)	8 (9.0)		0.495
Others, *n* (%)	3 (3.7)	2 (2.3)		0.584

In the NIRS group 89.0% (*n* = 73) and in the control group 94.4% (*n* = 84) (*p* = 0.202) were delivered by Caesarean section and 11.0% (*n* = 9) and 5.6% (*n* = 5) in the NIRS group and in the control group were delivered spontaneously, respectively. The time until cord clamping did not differ between the groups (*p* = 0.708). Cord clamping was performed at less than 30 s in 80.8% (*n* = 63) in the NIRS group and in 75.3% (*n* = 61) in the control group. The cord was clamped after 30 to 60 s in 3.9% (*n* = 3) in the NIRS group and in 4.9% (*n* = 4) in the control group, whereby cord clamping was delayed more than 60 s in 15.4% (*n* = 12) in the NIRS group and in 19.8% (*n* = 16) in the control group. Maternal and fetal causes for preterm birth are displayed in Table 1.

Provided respiratory support and medications during the first 15 min after birth and within the first 24 h after birth are presented in Table 2.

**Table 2:** Interventions during first 15 min after birth and the first 24 h after birth in very preterm neonates of the NIRS group and the control group

	NIRS group *n* = 82	Control group *n* = 89	*p*-value
*First 15 min after birth*			
Supplemental oxygen, *n* (%)	81 (98.8)	86 (96.6)	0.622
No respiratory support, *n* (%)	1 (1.2)	1 (1.1)	0.930
Mask continuous positive pressure, *n* (%)	23 (28.1)	29 (32.6)	
Mask positive pressure ventilation, *n* (%)	54 (65.9)	55 (61.8)	
Intubation, *n* (%)	4 (4.9)	4 (4.5)	
Chest compressions, *n* (%)	0 (0.0)	2 (2.3)	0.500
Caffeine, *n* (%)	42 (52.5)	53 (62.9)	0.171
Adrenaline, *n* (%)	0 (0.0)	0 (0.0)	-
Surfactant, *n* (%)	3 (3.7)	4 (4.5)	1.000
Volume, *n* (%)	3 (3.7)	0 (0.0)	0.108
Others, *n* (%)	4 (4.9)	2 (2.3)	0.433
*First 24 h after birth*			
Surfactant, *n* (%)	60 (73.2)	65 (73.0)	0.984
No respiratory support, *n* (%)	2 (2.4)	5 (5.6)	0.616
Non-invasive ventilation, *n* (%)	66 (80.5)	69 (77.5)	
Mechanical ventilation, *n* (%)	14 (17.1)	15 (16.9)	

### Primary outcome

Fidgety movements were analysed at 12.1 (11.9–12.7) weeks of corrected age in the NIRS group and at 12.1 (12.0–12.6) weeks in the control group (*p* = 0.819). No difference in the combined primary outcome (survival and normal fidgety movements between six to 20 weeks of corrected age) was observed comparing the NIRS group to the control group (RR [95%CI] 0.96 [0.31–2.62], *p* = 0.938) (Table 3).

**Table 3:** Combined outcome mortality and fidgety movements of very preterm neonates in the NIRS group and in the control group. Poor outcome is defined as mortality before 20 weeks of corrected age or pathological fidgety movements between six to 20 weeks of corrected age. Neonatal morbidities at term age or before discharge of very preterm neonates in the NIRS group and in the control group. Data are presented as n (%). Relative Risk [95% CI] and p-value of the Relative Risk are provided

	NIRS group *n* = 82	Control group *n* = 89	Relative Risk [95% CI]	*p*-value
Combined outcome mortality and fidgety movements				
Normal outcome	74 (90.2)	80 (89.9)	0.96 [0.31–2.62]	0.938
Poor outcome	8 (9.8)	9 (10.1)		
Neonatal morbidities at term age or before discharge				
Death and/or cerebral injury, *n* (%)	8 (9.8)	17 (19.1)	0.51 [0.23–1.12]	0.084
Death, *n* (%)	4 (4.9)	4 (4.5)	1.00 [0.93–1.06]	1.000
IVH any grade, *n* (%)	6 (7.3)	14 (15.7)	0.46 [0.19–1.15]	1.000
No IVH, *n* (%)	76 (92.7)	75 (84.3)		0.25
IVH I-II, *n* (%)	4 (4.9)	10 (11.2)		
IVH III-IV, *n* (%)	2 (2.4)	4 (4.5)		
Cystic PVL any grade, *n* (%)	1 (1.2)	1 (1.1)	1.09 [0.07–17.07]	1.000
No cystic PVL, *n* (%)	81 (98.8)	88 (98.9)		0.731
Cystic PVL II, *n* (%)	0 (0.0)	1 (1.1)		
Cystic PVL III, *n* (%)	1 (1.2)	0 (0.0)		
IRDS any grade, *n* (%)	77 (93.9)	84 (94.4)	0.99 [0.92–1.07]	1.000
No IRDS, *n* (%)	5 (6.1)	5 (5.6)		0.619
IRDS grade 1–2, *n* (%)	67 (81.7)	68 (76.4)		
IRDS grade 3–4, *n* (%)	10 (12.2)	16 (18.0)		
Culture proven sepsis, *n* (%)	19 (23.2)	23 (25.8)	0.90 [0.53–1.52]	0.164
NEC any grade, *n* (%)	3 (3.7)	1 (1.1)	3.26 [0.35–30.68]	0.351
BPD, *n* (%)	7 (8.5)	9 (10.1)	0.84 [0.32–2.16]	0.125
ROP grade ≥ 2, *n* (%)	7 (8.5)	11 (12.4)	0.69 [0.28–1.70]	0.463
PDA with interventions, *n* (%)	4 (4.9)	9 (10.1)	0.48 [0.15–1.51]	0.197

*BDP* bronchopulmonary dysplasia, *IRDS* infant respiratory distress syndrome, *IVH* intraventricular haemorrhage, *NEC* necrotizing enterocolitis, *PDA* persistent ductus arteriosus, *PVL* periventricular leukomalacia, *ROP* retinopathy of prematurity

### Secondary outcome

Eight preterm neonates died before 20 weeks of corrected age (*n* = 4 NIRS group; *n* = 4 control group). Survival without cerebral injury was 90.2% in the NIRS group and 80.9% in the control group (RR [95% CI] 1.12 [0.99–1.26], *p* = 0.084). Secondary outcomes including IVH, cystic PVL, IRDS, EOS, NEC, BPD, ROP and PDA with interventions are displayed in Table 3.

## Discussion

This is the first study, investigating the potential influence of cerebral oxygenation guided resuscitation during immediate fetal-to-neonatal transition period on combined outcome of mortality and fidgety movements defined as normal or pathological in very preterm neonates. We did not observe substantial impact on mortality and fidgety movements in the NIRS group when compared to the control group.

Pathological fidgety movements, especially their absence, have been described as a predictive value for a later development of cerebral palsy [23, 24]. Whereby, a combination of both, cerebral injury and absence of fidgety movements have the highest predictive value. Abnormal GMs or absence of fidgety movements in preterm neonates with cerebral morbidities, especially PVL, are explained by disruptions of the corticospinal projection as a consequence of brain lesions [25, 26]. In our present cohort, the proportion of analysed preterm neonates with cerebral injuries was quite low. Therefore, the overall risk for the development of a cerebral palsy in our observed cohort is very low, and it may be speculated that the results of the present study may be more pronounced in cases of a higher proportion of cerebral injuries.

Beside the high predictive value for cerebral palsy in case of absent fidgety movements, no significant association of abnormal/absent fidgety movements and Bayley Scales of Infant and Toddler Development-Third Edition (BSID-III) has been described [27]. The potential influence of crSO_2_ on long-term outcome at a corrected age of two years, assessed by BSID-III has been observed by Wolfsberger et al. [28]. They stated that preterm neonates with a very low gestational age and birth weight with poor long-term outcome (mortality, testing not possible due to cognitive impairment and/or BSID-III ≤ 70), showed significant lower crSO_2_ values during immediate fetal-to-neonatal transition period, when being compared to preterm neonates with favourable outcome.

The results of fidgety movements between six to 20 weeks of corrected age can be influenced by different perinatal factors and morbidities. The predictive value of fidgety movements also depends on the postnatal age / corrected age of the neonate at time point of assessment [25]. The mean corrected age at time point of assessment of fidgety movements of the included preterm neonates in our cohort was 12 weeks. According to literature, the optimal period for evaluating fidgety movements is generally regarded between ten to 12 weeks post-term [8]. Based on that, results of fidgety movements demonstrated in the present study were assessed in the ideal period concerning evaluation of neurobehavioral repertoire. Beside postnatal age, severe infections during early neonatal period may have an impact on fidgety movements, with a higher proportion of abnormal/absent fidgety movements being observed in neonates with infections [29]. In the present study no difference in culture proven sepsis was described between the two groups. Therefore, the similar distribution of this influencing factor can be reassuring for interpretation of the present results. Furthermore, a retrospective study described a higher percentage of abnormal fidgety movements in very preterm neonates who received systemic corticosteroids compared to neonates of the non-corticosteroid group [30]. In our present study, unfortunately no information about therapy with systemic corticosteroids were available. Gestational age and severity of illnesses, however, were similar between the two groups suggesting also similar corticosteroid application.

It has been described that serial GMAs provide a more accurate information about the early neurodevelopmental state of the analysed neonate [31, 32]. Changes in the quality of the GMs, can be influenced by different factors, including provided medications or interventions, as described above. Multiple GMAs at different time points might help to rule out these potential influencing factors [31, 32]. In our present cohort of neonates, serial GMAs have only been performed in a few preterm neonates, therefore serial GMA analysis was not possible, which might be a limiting factor.

Taking the results of the present study into account it may be assumed that crSO_2_ monitoring with dedicated interventions according to the COSGOD III trial cannot influence mortality up to 20 weeks and the appearance of fidgety movements between six to 20 weeks of corrected age in very preterm neonates. This is in contrast to already published data observing the potential influence of burden of cerebral hypoxia within the first 15 min after birth on GMA, and GMOS performed between 36 and 40 weeks postmenstrual age [14]. These results suggest that cerebral oxygenation during immediate fetal-to-neonatal transition period affect GMs at term age. Pansy et al. [14] suggested burden of hypoxia defined by crSO_2_ values below the 10th percentile [33] might have an influence on cerebral injury and thus on neonatal neurodevelopmental outcome. Different assumptions may explain the discrepancy between our present findings to already published data describing an influence of crSO_2_ on short-term neurological outcome. Writhing movements, as investigated in the study of Pansy et al. [14], and fidgety movements cannot be equated. Souza et al. [34] observed potential correlation between writhing movements and fidgety movements in context to assess the time point when writhing movements are predictive for fidgety movements. They described that abnormal writhing movements at the neonatal intensive care unit or up to five weeks of corrected age, were only in 85% in accordance to absent fidgety movements nine to 20 weeks of corrected age.

The major strength of the study is the amount of overall included preterm neonates with available fidgety movements (*n* = 163) of a multicentre study. The main limitation of this study was the overall number of analysed preterm neonates with abnormal/absent fidgety movements. This, however, may be explained by the overall low proportion of preterm neonates with a cerebral injury, especially with a diagnosis of cystic PVL.

## Conclusion

Cerebral oxygen saturation monitoring combined with dedicated treatment guidelines in accordance to the protocol of the COSGOD III trial during immediate fetal-to-neonatal transition period was not associated with an improvement on combined outcome, death and/or pathological fidgety movements between six to 20 weeks of corrected age.

## Data Availability

Data are available on reasonable request.

## Abbreviations

BSID-III: Bayley Scales of Infant and Toddler Development-Third Edition
BPD: Bronchopulmonary dysplasia
crSO_2_: Cerebral oxygen saturation
GMA: General Movement Assessment
GMOS: General movement optimality score
GMs: General Movements
IRDS: Infant respiratory distress syndrome
IVH: Intraventricular haemorrhage
NIRS: Near-infrared spectroscopy
NEC: Necrotizing enterocolitis
PVL: Periventricular leukomalacia
PDA: Persistent ductus arteriosus
ROP: Retinopathy of prematurity
